# Retraction Note to: IRE1α-XBP1 but not PERK inhibition exerts anti-tumor activity in osteosarcoma

**DOI:** 10.1007/s12672-022-00481-6

**Published:** 2022-03-25

**Authors:** Keita Sasa, Tsuyoshi Saito, Taisei Kurihara, Nobuhiko Hasegawa, Kei Sano, Daisuke Kubota, Keisuke Akaike, Taketo Okubo, Takuo Hayashi, Tatsuya Takagi, Takashi Yao, Muneaki Ishijima, Yoshiyuki Suehara

**Affiliations:** 1 Department of Medicine for Orthopaedics and Motor Organ, Juntendo University School of Medicine, Tokyo, Japan; 2 Department of Human Pathology, Juntendo University School of Medicine, Tokyo, Japan; 3 Intractable Disease Research Center, Graduate School of Medicine, Juntendo University, 2-1-1, Hongo, Bunkyo-ku, Tokyo, 113-8421 Japan

## Retraction to: Discover Oncology (2021) 12:57 10.1007/s12672-021-00453-2

The Editor in Chief has retracted this article. Concerns were raised after publication, and subsequent post publication peer review concluded that there was insufficient experimental work for this article. Therefore, the Editor in Chief has lost confidence in the results of the study. The authors have been offered to submit a revised manuscript for further peer review.

Tsuyoshi Saito, Keita Sasa, Taisei Kurihara, Daisuke Kubota, and Muneaki Ishijima agree to this retraction. Nobuhiko Hasegawa, Kei Sano, Keisuke Akaike, Taketo Okubo, Takuo Hayashi, Tatsuya Takagi, Takashi Yao, and Yoshiyuki Suehara have not responded to any correspondence from the editor about this retraction.

